# Negative Index Metamaterial-Based Frequency-Reconfigurable Textile CPW Antenna for Microwave Imaging of Breast Cancer

**DOI:** 10.3390/s22041626

**Published:** 2022-02-18

**Authors:** Kabir Hossain, Thennarasan Sabapathy, Muzammil Jusoh, Shen-Han Lee, Khairul Shakir Ab Rahman, Muhammad Ramlee Kamarudin

**Affiliations:** 1 Advanced Communication Engineering (ACE), Centre of Excellence, Universiti Malaysia Perlis (UniMAP), Jalan Tiga, Pengkalan Jaya Business Centre, Kangar 01000, Malaysia; hossain.kabir42@gmail.com (K.H.); muzammil@unimap.edu.my (M.J.); 2 Faculty of Electronic Engineering Technology, Universiti Malaysia Perlis (UniMAP), Kampus Alam UniMAP Pauh Putra, Arau 02600, Malaysia; 3 Department of Otorhinolaryngology, Hospital Sultanah Bahiyah, KM6 Jalan Langgar, Alor Setar 05460, Malaysia; shen-han.lee@cantab.net; 4 Department of Pathology, Hospital Tuanku Fauziah, Kangar 01000, Malaysia; ksyakir@gmail.com; 5 Faculty of Electrical and Electronic Engineering, Universiti Tun Hussein Onn Malaysia (UTHM), Parit Raja, Batu Pahat 86400, Malaysia; mramlee@uthm.edu.my

**Keywords:** metamaterials, textile antennas, reconfigurable textile antennas, cancer, breast cancer, microwave imaging

## Abstract

In this paper, we report the design and development of a metamaterial (MTM)-based directional coplanar waveguide (CPW)-fed reconfigurable textile antenna using radiofrequency (RF) varactor diodes for microwave breast imaging. Both simulation and measurement results of the proposed MTM-based CPW-fed reconfigurable textile antenna revealed a continuous frequency reconfiguration to a distinct frequency band between 2.42 GHz and 3.2 GHz with a frequency ratio of 2.33:1, and with a static bandwidth at 4–15 GHz. The results also indicated that directional radiation pattern could be produced at the frequency reconfigurable region and the antenna had a peak gain of 7.56 dBi with an average efficiency of more than 67%. The MTM-based reconfigurable antenna was also tested under the deformed condition and analysed in the vicinity of the breast phantom. This microwave imaging system was used to perform simulation and measurement experiments on a custom-fabricated realistic breast phantom with heterogeneous tissue composition with image reconstruction using delay-and-sum (DAS) and delay-multiply-and-sum (DMAS) algorithms. Given that the MWI system was capable of detecting a cancer as small as 10 mm in the breast phantom, we propose that this technique may be used clinically for the detection of breast cancer.

## 1. Introduction

Female breast cancer is the most commonly diagnosed cancer worldwide, recently surpassing even lung cancer, with 2.3 million new cases in 2020 [1]. Early detection and diagnosis of this disease is the most effective strategy to reduce mortality, since patients diagnosed at an early stage of cancer have the best chance for curative treatment and long-term survival [2]. In low- and middle-income countries with lesser health resources, a larger proportion of women with breast cancer present late and are ultimately diagnosed with locally advanced or metastatic disease [3]. This gives rise to a wide disparity in the breast cancer survival rates between high-income versus low- and middle-income nations [1]. Hence, the importance of early breast cancer detection in low-resource settings cannot be overstated.

Current medical imaging modalities in breast cancer include mammography, ultrasonography, computed tomography (CT), and magnetic resonance imaging (MRI). These diagnostic tools have limitations and, therefore, there is an unmet need to explore alternative or complementary tools and techniques for early detection of breast cancers [4]. Although mammography is the current gold standard diagnostic and screening tool for breast cancer, it suffers from several shortcomings, including the discomfort from breast compression, and reduced sensitivity in cancer detection under certain circumstances, such as in cancers without a definite mass (e.g., in invasive lobular breast cancers), without calcification, and in breasts of a higher density. While ultrasound scan is portable and cost-effective, the major drawback of this technique is that it is operator-dependent and has poor specificity. CT and MR imaging lack portability and come at a considerable cost, limiting their use in low-resource settings. In addition, the use of ionising radiation in mammography and CT imaging may result in harmful health effects such as increased risk of developing cancer with excessive cumulative exposure over time.

Microwave imaging is an emerging technique in early breast cancer detection. It has been proposed as a safe, mobile, and cost-effective adjunct to conventional imaging modalities. Low cost and portability of the microwave hardware system also make it ideal for use in low-resource settings. The performance of a microwave imaging hardware system depends on the antenna which transmits microwaves and receives backscattered signals from the irradiated object [5]. A higher antenna frequency results in improved image resolution but comes at a cost of shorter wavelength and shallower depth of penetration, precluding the detection of small, deep-seated cancers. The optimum frequency for the MWI system for tissue sensing is still a subject of investigation, with no consensus on a particular bandwidth that is best suited for MWI. However, Bahramiabarghouei et al. reported achieving optimum results between 2 and 4 GHz [6]. Nikolova et al. [7] reported that a microwave frequency range from 0.5 GHz to 3 GHz is the best choice in order to achieve deeper tissue penetration with an acceptable imaging quality. In addition, the high permittivity of the human body causes a frequency shift when the antenna is placed near the body. This problem of mutual coupling between the antenna and human body is especially pertinent for wearable antennas. Other sources of frequency shifts may arise from fabrication error and physical deformation in the case of flexible or wearable antennas [8].

Several technical modifications and refinements to antenna design have been proposed to improve its performance for microwave imaging. Work carried out over the past decade have employed ultrawideband (UWB) antenna technology, with its unique features of high-speed data rates, very small signal interference, simple low-cost design, to construct microwave imaging systems [5]. Existing work [9,10] have also explored the use of flexible materials to design the antennas and, thus, it will be suitable for on-body applications. In addition, previous studies demonstrated that incorporating metamaterials (MTMs) into conventional antenna design resulted in enhancement of antenna’s gain bandwidth, higher radiation efficiency, improvement in radiation pattern, and reduction of specific absorption rate (SAR) [8,11,12]. MTMs are artificially-engineered structures which exhibit non-naturally occurring electric and magnetic properties such as negative refractive index [13]. The MTMs usually comprise an array of structures, termed unit cells, that are smaller than the wavelength of interest and can be characterised by their magnetic permeability (*μ*) or dielectric permittivity (*ε*). Numerous metamaterial structures have been suggested, including planar patterns, split-ring resonators (SRRs) [14,15], capacitance-loaded strips (CLSs), and complementary split-ring resonators (CSRRs) [16,17]. Other than that, several MTM structures exist, including artificial magnetic conductors (AMCs) and electromagnetic bandgaps (EBGs) [18]. MTMs have been incorporated in UWB-wearable antennas as part of microwave imaging systems to detect melanoma [19] and breast cancer [20].

Most MTMs-based antennas are static, and their electromagnetic properties cannot be controlled once fabricated, resulting in high losses, narrow bandwidth, and tolerance sensitivity. In contrast, the ability to reconfigure or tune MTM-based antennas has been shown to overcome most of these limitations [8,21]. The reconfigurability of such antennas allow tuning of their frequency, pattern, polarization, or a combination of properties, thereby permitting operation across several frequency bands using a single piece of hardware that is both cost-effective and compact [22,23,24,25].

Reconfigurable/tunable antennae can tune frequency, pattern, and polarisation, or a combination of these. A frequency reconfigurable antenna is a type of antenna that can operate across several frequency bands using a single piece of hardware that is both cost-effective and compact [22,23,24,25]. Apart from that, when more concerns are directed to the flexible/textile wearable antenna, it should be kept in mind that after fabrication, the result of the measurements would shift to a higher or lower frequency due to fabrication error, physical deformation, or other well-known reasons evidenced in many studies [8]. After careful consideration, varactor diodes are adopted in this study to achieve the tunability feature since the antennas are aimed at tuning a particular frequency region.

Previous works using MTM-based antennas for MWI in breast cancer detection are summarised in Table 1 in terms of antenna type, antenna size, tunability feature, operating frequency, frequency/time-domain adopted into the study, imaging algorithms, types of the phantom, number of cancer/s incorporated in the breast phantom, and type of substrate used in antenna design. Thus far, no study has employed reconfigurable antennas, either in a flexible or rigid format, for the use in microwave imaging studies of breast cancer detection. Apart from that, no study has investigated the coupling effect of human tissue (and/or mimicked tissue), which results in the frequency shifting during microwave imaging experiments.

This study was aimed at focusing on development of a negative-index MTM-based frequency-reconfigurable antenna for breast cancer detection systems for the first time, to the best of the authors knowledge, to contribute to the technological advancement related to breast cancer detection system. Based on proper justification and literature review, in this study, the 3 GHz frequency band was chosen to design the proposed antenna and to carry out investigation on the microwave breast imaging system. The key contribution of this study was the development of a negative index MTM-based directional coplanar waveguide (CPW)-fed frequency-reconfigurable textile antenna with adopting RF varactors. The directional radiation pattern produced by the proposed antenna aids breast cancer imaging by reducing unwanted signal from the back lobe of the antenna. On the other hand, the frequency reconfigurability provided the flexibility to retune the antenna frequency and, hence, maintained the performance of the antenna. Overall, the MTM-based reconfigurable antenna played an important role to maintain a high-quality data creation which is of paramount important not only in the study of a breast imaging system but also in any kind of imaging system. Moreover, a realistic heterogeneous lab-made breast phantom was fabricated and experimentally validated prior adopting to the proposed MTM-based frequency reconfigurable textile antenna driven breast imaging system investigation. Although different imaging algorithms have been adopted in various studies, the recent experimental findings in [32] confirmed that delay-and-sum (DAS) and delay-multiply-and-sum (DMAS) algorithms provide consistent results compared to other state-of-the-art imaging algorithms. Furthermore, based on literature survey and careful observations, DAS and DMAS algorithms were adopted to carry out this study.

## 2. Overview of Workflow

**Figure 1:**
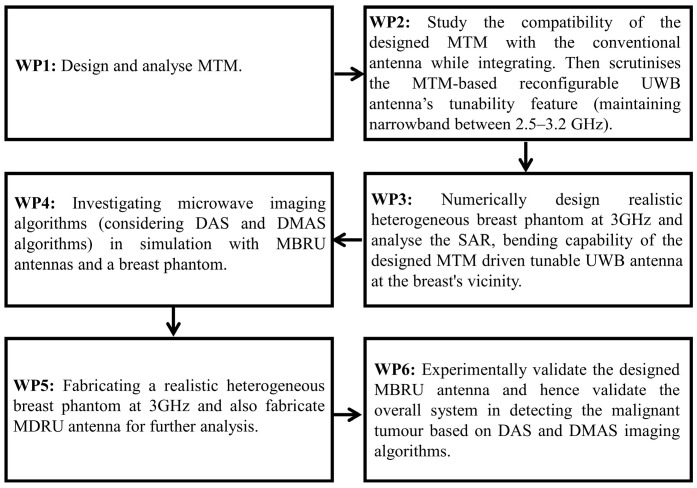
Block diagram of research phases in this study.

Figure 1 depicts the overall research flow of this study, and it was completed in six (6) work packages (WPs). We began by designing and characterising the textile-based MTM in WP1. In WP2, we integrated the MTM with a conventional antenna to produce an MTM-based reconfigurable antenna (MBRU) with the aid of RF varactor diodes. The tunability feature was measured, maintaining narrowband between 2.5–3.2 GHz. In WP3, a breast phantom with heterogeneous tissue composition was designed, and the compatibility of the proposed antenna in terms of SAR and bending were analysed using simulations. In WP4, investigations were carried out utilising microwave imaging algorithms, DAS and DMAS algorithms, with the aid of designed MBRU antenna and breast phantom. The breast phantom and the proposed MBRU antenna were then fabricated in WP5 for further investigation. Finally, in WP6, the overall MWI system was experimentally validated for breast imaging and cancer detection in the breast phantom using the DAS and DMAS imaging algorithms.

## 3. Metamaterial-Based Reconfigurable Antenna Design

The MTM and the MTM-based reconfigurable textile antennas were modelled and simulated using Computer Simulation Technology (CST) Microwave Studio Suite (MWS). The CST MWS was utilised to simulate the MTM unit cell, and MATLAB was subsequently employed to extract the material effective parameters (permittivity, refractive index) from the MTM’s S-parameter.

Other than simulating the proposed MBRU antenna, fabrication was also carried out using textile materials. Shieldit Super^TM^ was utilised as the ground plane and radiator. It has a thickness of 0.17 mm and a conductivity of 1.18 × 10^5^ S/m. Three-millimetre-thick felt substrate with 1.44 dielectric constant and 0.044 loss tangent was used as a dielectric material.

In designing the MTM, several factors are taken into consideration. The dimensions of the MTM mainly depend on the wavelength of the application, the bandwidth of interest, the material, and manufacturing capabilities, as well as its limitations. Additionally, different structures give different MTMs effective parameters and it is considered as a new MTM, where the structures can be symmetric or asymmetric and isotropic or anisotropic [33]. Moreover, the MTM unit cell dimension should be smaller than 1/10 of the operating wavelength [13,33,34]. Since the wavelength (*λ*) is equal to the product of the speed of light (*v*) divided by the frequency of light (*f*), (*λ* = *v*/*f*)*,* a metamaterial with a dimension of 1 cm has its resonance at 4 GHz, which is roughly 1/10 of a wavelength. Although the electromagnetic properties of interest may be far from its resonance frequency, the negative values for *µ* and/or *ε* are close to the resonance frequency.

**Figure 2:**
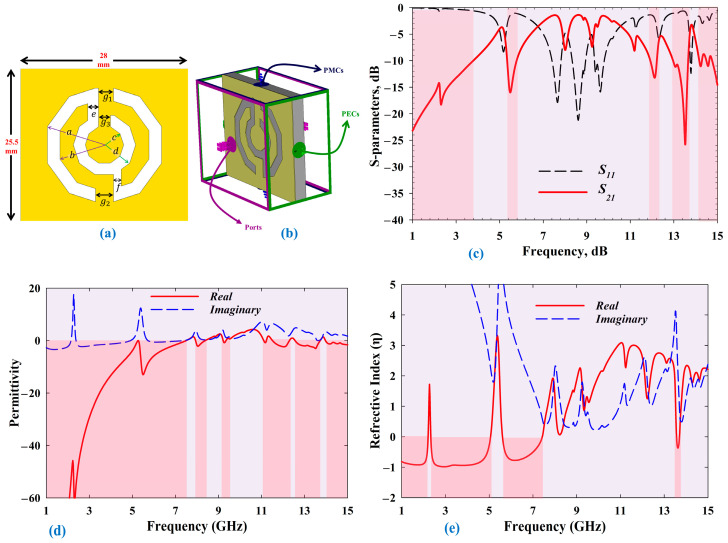
MTM unit cell model and simulated results under WP1: (**a**) schematic of the proposed unit cell, (**b**) 3D view of the MTM simulation setup, (**c**) simulated S-parameters, (**d**) permittivity, and (**e**) refractive index.

In this study, the designed MTM was based on several pairs of symmetric decagonal C-shaped complementary split-ring (CSRR) resonators that were assembled and cover each other to enable the unit cell to operate over a wide frequency range—the design of the MTM is shown in Figure 2a. Furthermore, all physical parameters of the MTM unit cell design are summarised in Table 2. To determine the effective parameters of the MTM, the structure was placed between two waveguide ports on the positive and negative *z*-axis. A transverse electromagnetic (TEM) wave was used for this, as shown in Figure 2b. Perfect electric conductor (PEC) boundaries were used to define the *x*-axis and perfect magnetic conductor (PMC) boundaries were used to define the *y*-axis of the model, respectively. The CST MWS simulator with a frequency-domain solver with a tetrahedral mesh was used across the frequency range of 1 to 15 GHz to extract the material’s effective parameters. The simulated result of the designed MTM is presented in Figure 2c–e, where the negative index region is highlighted in red. Simulated results revealed that the attainable *S*_21_ bandwidth (BW) was 4.608 GHz, permittivity BW was 10.5 GHz, and refractive index BW was 5.99 GHz. More details pertaining to this MTM unit cell design and analysis can be found in [35]. For the next, this MTM was adopted to design the MBRU antenna which was classified under WP2.

**Figure 3:**
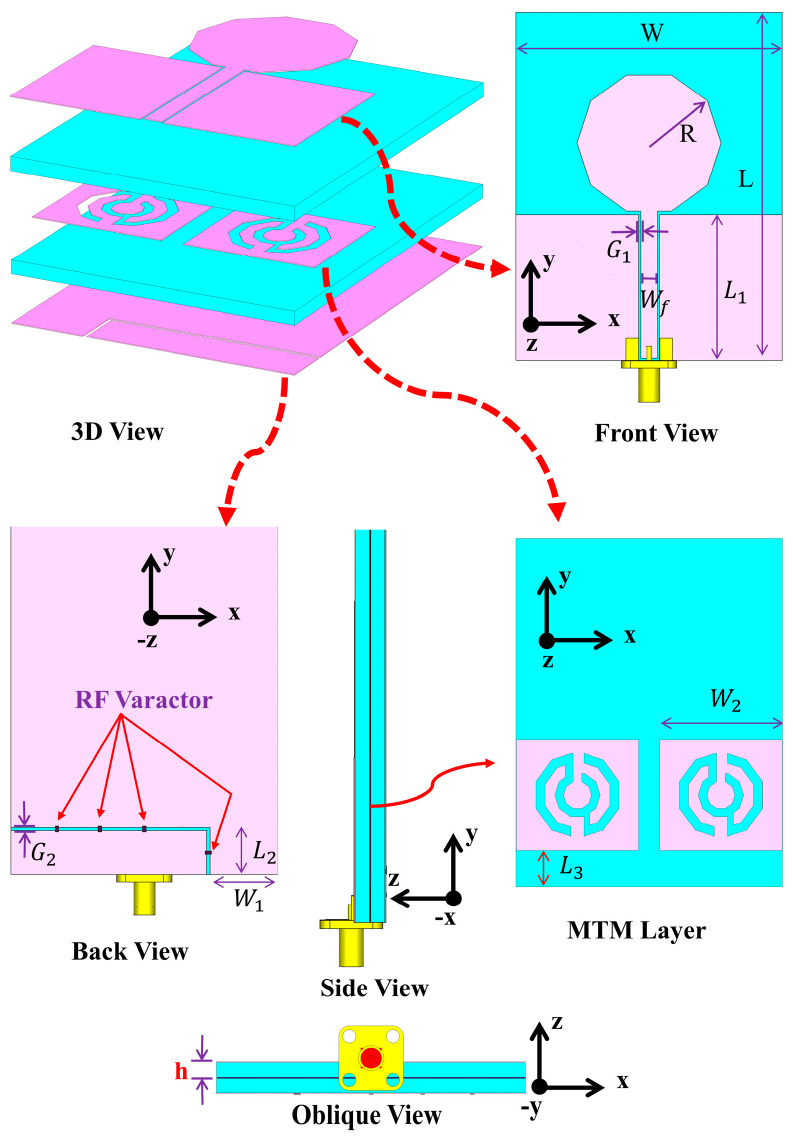
Schematic diagram of the MBRU antenna including the MTM layer.

Figure 3 depicts the MBRU antenna formed from the integration of a typical directional CPW-fed antenna with the decagonal C-shaped CSRR unit cells. The CPW-fed antenna in this study was designed with a decagonal shape patch. A full ground plane was directly added as a reflector under the substrate layer. A unidirectional radiation pattern was obtained this way, but the bandwidth of the impedance was significantly reduced [36]. To maximise the bandwidth and retain a relatively compact form factor and thickness, the MTM was added underneath the radiating patch layer. A 50-Ω SMA connector was connected at the end of the feedline. The overall dimension of the antenna is 80 × 61 × 6 mm^3^ (0.8 λ_0_ × 0.61 λ_0_ × 0.06 λ_0_), where λ_0_ is the free space wavelength at 3 GHz, with the MTM unit cell sized at 28 × 25.5 mm^2^ (0.28 λ_0_ × 0.255 λ_0_). All dimensions are summarised in Table 3.

**Figure 4:**
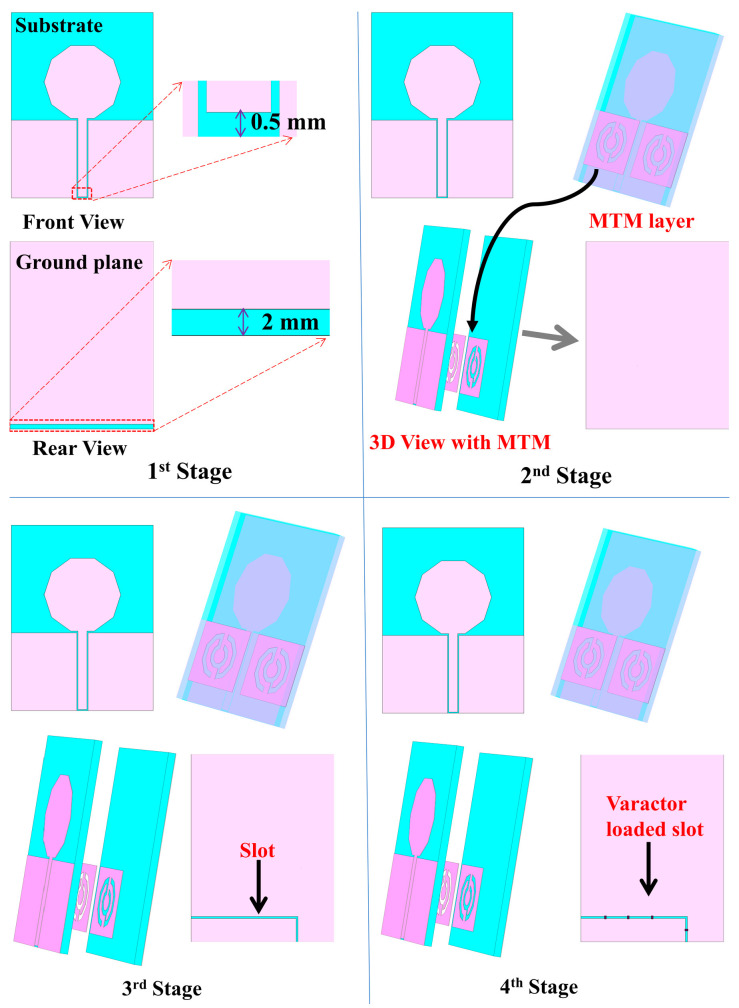
Evolution process of the MBRU antenna in this study.

Figure 4 depicts the evolution process of the MBRU antenna design in this study. In the first stage, a full ground patch with a directional CPW-fed decagonal-shaped patch-type conventional antenna was designed with a 0.5 mm gap to avoid short circuit between the 50 Ω connector and the feedline of the antenna. To achieve wider impedance bandwidth, MTM was added beneath the patch antenna layer, as illustrated in the second stage. To achieve tunability of the MTM-driven antenna, an L-shaped slot was added in the third stage in the reflector ground plane. The RF varactor diodes (SMV1232-079LF, Skyworks, Irvine, CA, USA) were then added at the L-shaped slot in the fourth stage to avail the tunability feature. The working principle of the RF varactor diode is described in [21].

### 3.1. Results of the Proposed MBRU Antenna

One of the crucial features of the reconfigurable antenna is to enable efficient tuning to a targeted parameter while simultaneously maintaining optimum performance in the characteristics of other parameters [22,37]. For example, a frequency-reconfigurable antenna was previously reported where it produced directional radiation patterns at higher frequencies and a quasi-omnidirectional radiation pattern at lower frequencies [23].

**Figure 5:**
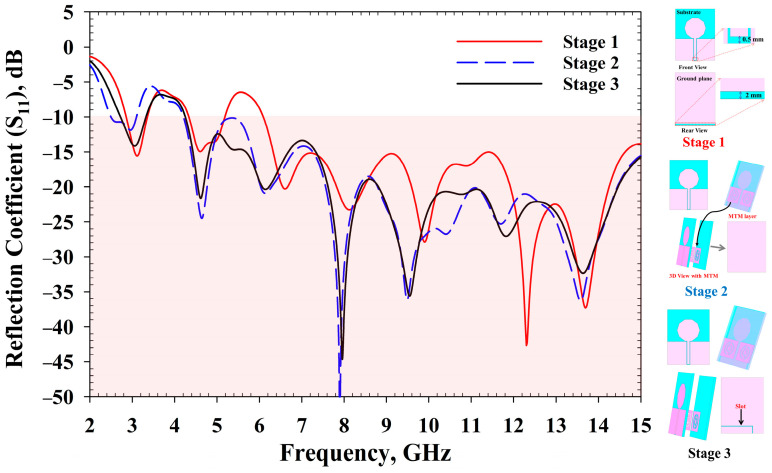
Effects of MTM and evolution of MBRU.

The effects of MTM and the evolution of the desired antenna are shown in Figure 5. Firstly, a decagonal-shaped patch was introduced in stage 1, and was CPW-fed. Although the calculated size and radius of the patch suggested that this antenna theoretically operates at 8.5 GHz, a lower operating frequency of 3.4 GHz was observed. However, the bandwidth was improved with the integration of MTM with the antenna in stage 2, and static frequency bands of 4–15 GHz were attained. These results demonstrated that the antenna resonant frequency can be modulated by integrating the MTM. The effect of the MTM unit cells on the conventional antenna can be observed by comparing *S*_11_ results between stage 1 and stage 2. This may be due to impedance mismatching at a lower frequency, especially at 6 GHz antenna in the absence of MTM integration. Likewise, a good impedance matching and wider bandwidth were gained with the use of two MTM unit cells. Subsequently, an L-shaped slot was integrated at the ground plane in stage 3 to ensure the antenna could resonate with controllable frequency at the lower frequency region of the Federal Communications Commission (FCC) allocated UWB region [38]. In addition, to maintain a directional radiation pattern, a full ground/reflector was implemented. The slot availability, assumed to be the switches or varactors deployed, operates at OFF state. Meanwhile, the ON condition is considered when the slot is closed using switches at ideal ON state. A simple DC biasing implementation strategy was adopted with minimal structural modification at the narrow slot when emplaced with varactors. Varactor diodes were adopted in this work instead of PIN diodes since varactors allow frequency tuning throughout a more comprehensive frequency range. This was achieved via modulation of their capacitance and isolation loss when reverse bias voltages were applied [39]. However, such configuration does not typically allow tuning with large frequency ratios [40,41,42].

**Figure 6:**
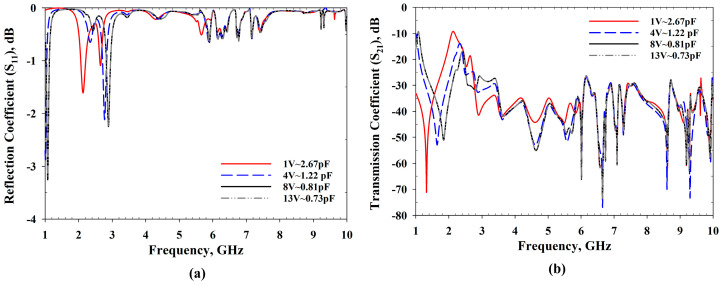
Effect of varactors tuning over MTM features: (**a**) *S*_11_ and (**b**) *S*_21_.

**Figure 7:**
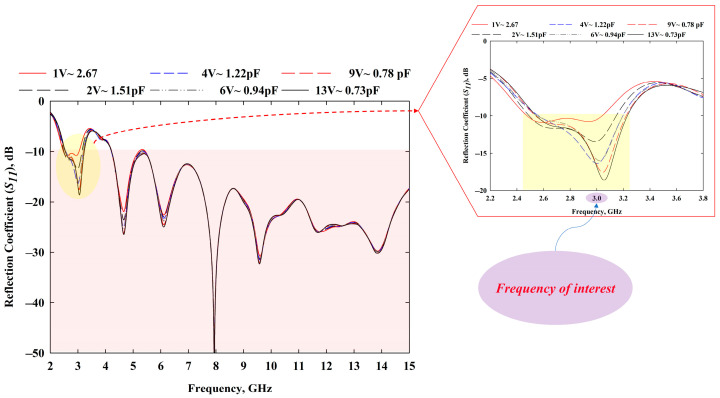
Simulated reflection coefficient, *S*_11_ results.

The MBRU antenna design process was completed in stage 4, where RF varactors were utilised to avail tunability features. The MTM was integrated to enhance the bandwidth of the design, as described earlier. From Figure 5, it is evident that before the MTM integration, the frequencies from 5.15 to 6.1 GHz were not lower than −10 dB, but the bandwidth was improved at stage 2. Overall, the reconfigurability feature adopted with the MTM allowed the antenna frequency to be tuned. The MTM parameters were also tuned with the use of varactors configuration (see Figure 6). The integration of the MTM not only contributes to the bandwidth improvement but also helps to tune the frequency of the antenna at the desired frequency range (2.5–3.2 GHz). Specifically, the MBRU antenna’s tuning region approximately follows the MTM reconfigurability features in terms of *S*_11_ of the unit cell with the use of RF varactors tuning. The tuning information of MTM and MBRU antenna can be seen in Figure 6 and Figure 7, respectively.

**Figure 8:**
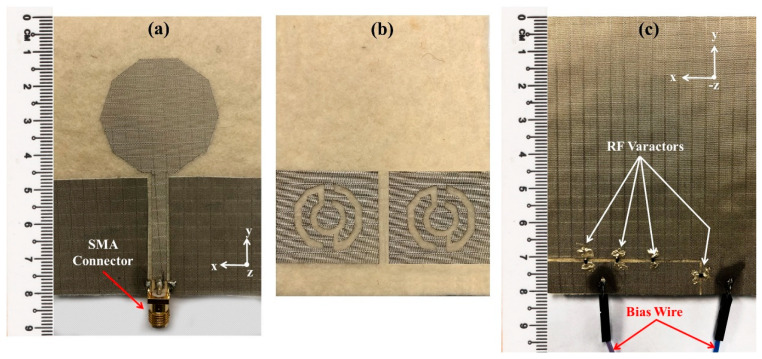
Fabricated prototype of the MTM driven reconfigurable antenna. (**a**) Top layer, (**b**) MTM layer, and (**c**) bottom layer.

**Figure 9:**
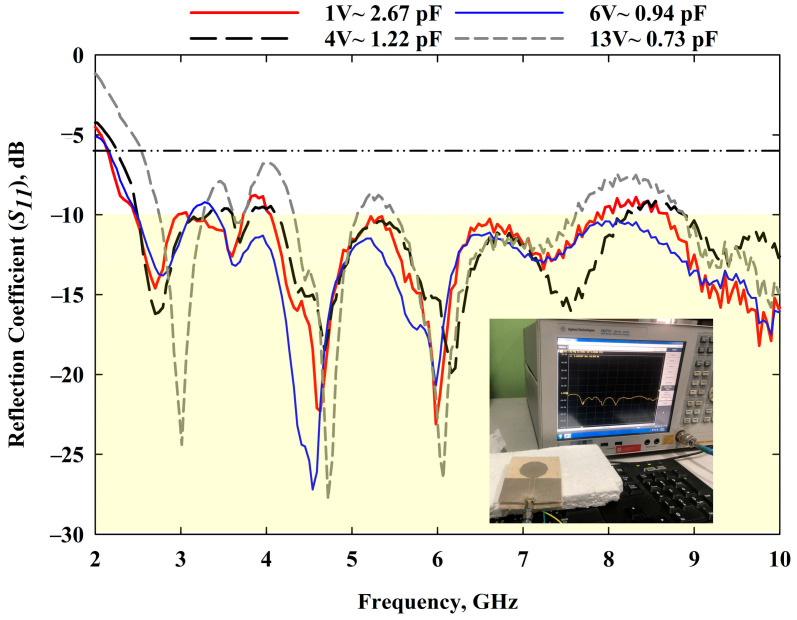
Measured *S*_11_ results.

The simulated reflection coefficients were obtained from the CST software, whereas measured *S*_11_ were obtained from an E5071C network analyser (Agilent Technologies, Bayan Lepas, Penang, Malaysia). The simulation results are presented in Figure 7, which was part of stage 4 described in Figure 4, whereas the experimental *S*_11_ of the presented prototype in Figure 8 (which was completed in WP5) is shown in Figure 9 (carried out in WP6). In all the operating frequency bands of the tuning region, the simulated and measured *S*_11_ are less than −10 dB. A minimal variation in frequency bands between simulation and measurement results was caused by the additional resistance introduced by the RF varactor diodes and biasing. The measured resonant frequencies were slightly shifted downwards for all operating bands with an average shift of about 40 MHz. During *S*_11_ measurements, the prototype was biased using an E3631A power supply (Keysight Technologies, Bayan Lepas, Penang, Malaysia). Despite this, the use of the varactor allowed slight retuning of the resonant frequency by marginally varying the applied reverse bias voltages. For instance, the measured resonant frequency of 3 GHz could be obtained with a reverse bias voltage of 11 V. Regardless of tuning region, the antenna can be applied for UWB applications as the 6 dB reflection coefficient is frequently used in the manufacturer’s specification, as reported in [43,44]. Hence, the obtained performance is sufficient for practical applications.

**Figure 10:**
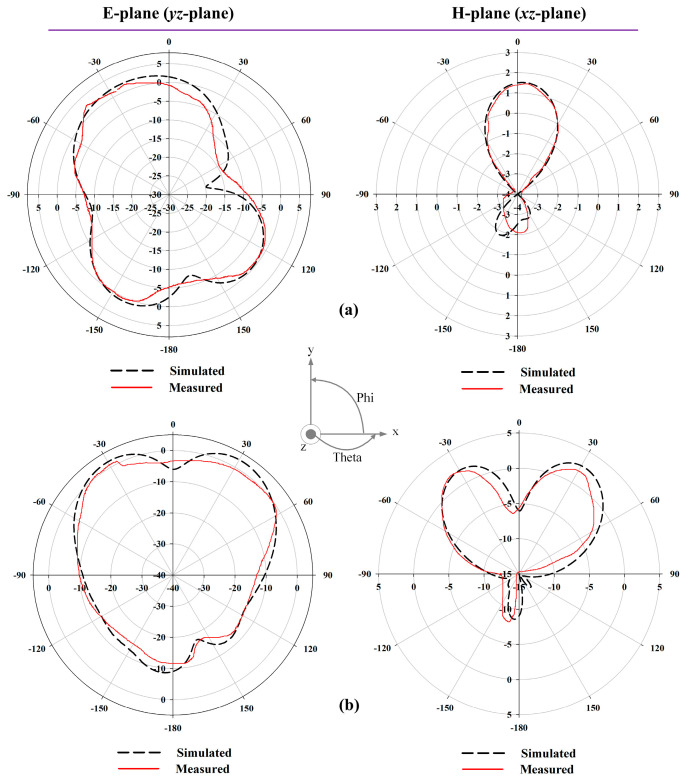
Simulated and measured normalised polar radiation pattern at biasing condition of 13 V (~0.73 pF): (**a**) 3 GHz and (**b**) 6.5 GHz.

**Figure 11:**
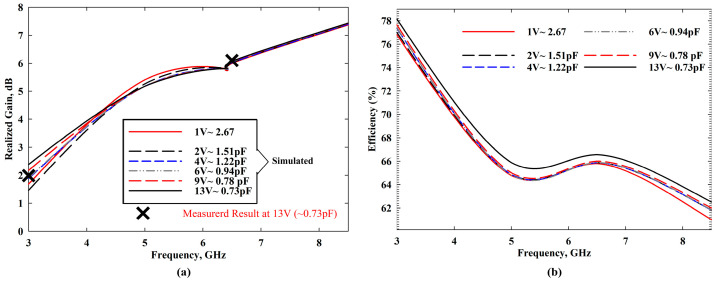
The proposed antenna’s (**a**) realized gain and (**b**) simulated efficiency.

The simulated and measured radiation patterns of the proposed antenna for applied reverse bias voltages at 13 V (~0.73 pF) are shown in Figure 10, where the measurements were carried out under WP6. The radiation patterns at 3 GHz and 6.5 GHz for E-plane (*yz*-plane) and H-plane (*xz*-plane) were directional and bidirectional, respectively. Moreover, Figure 11a shows the antenna gain and Figure 11b exhibits total efficiency over the frequency. The proposed antenna had a peak gain of 7.56 dBi—with a better gain at higher frequencies—and an average efficiency of more than 67%, supporting the utility of this antenna for microwave breast imaging.

#### 3.1.1. Study on Bending Analysis and SAR

**Figure 12:**
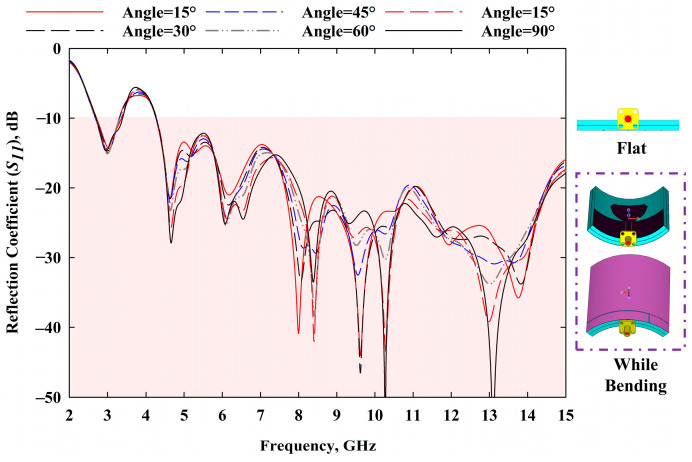
MBRU antenna bending analysis (at 4V varactor tuning condition).

The length of the proposed antenna is more likely to be bent along the *x*-axis in practical application, as previously described in a similar scenario [45]. Results of the analysis pertaining to physical deformation under varactor tuning conditions at 4 V are presented in Figure 12. All the conditions are presented, inclusive of MTM and the bending radii between 15° and 90° with a step width of 15°. While the return loss at the lowest operating frequency was barely changed, the return loss at 5.5 GHz improved with a decline of the bending arc radius. Taken together, these results suggest that even under deform conditions of the antenna, the impedance bandwidth was able to maintain a satisfactory outcome at the frequency of interest.

**Figure 13:**
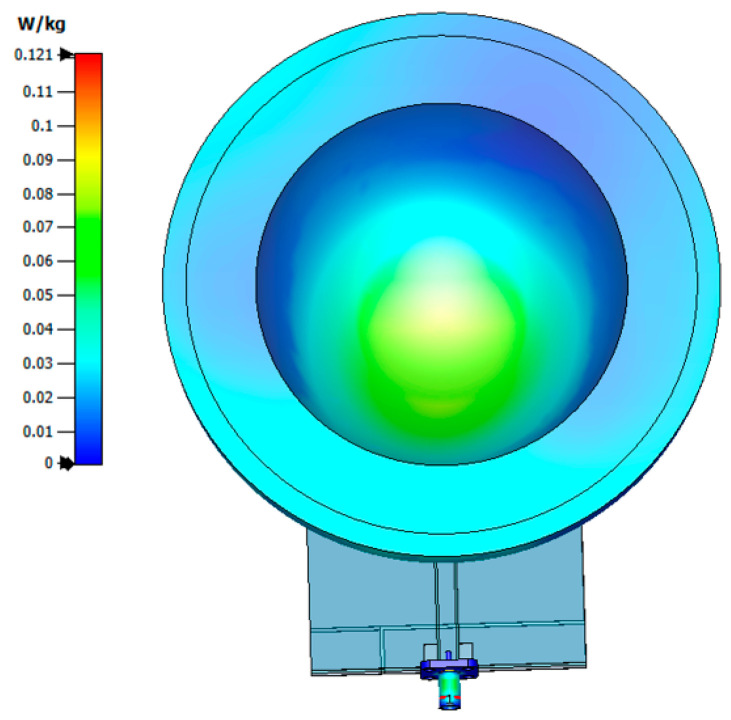
SAR distribution in the vicinity of the breast phantom of the MBRU antenna at 3 GHz under 10 g standard calculation.

Since overexposure to RF energy may be potentially harmful, the effects of the antenna radiation close to biological tissues were further studied at 3 GHz, using a 10 g tissue as a standard (Figure 13). The SAR maximum radiation concentration of the MBRU antenna is 0.121 W/kg, which meets the European standard of not more than 2.0 W/kg. These findings suggest that the developed MTM-based antenna can focus RF energy onto specific areas, while reducing the overall levels of RF exposure elsewhere, making it suitable as a wearable application for MWI.

#### 3.1.2. Effect of the MBRU Antenna in the Vicinity of the Human Tissue

**Figure 14:**
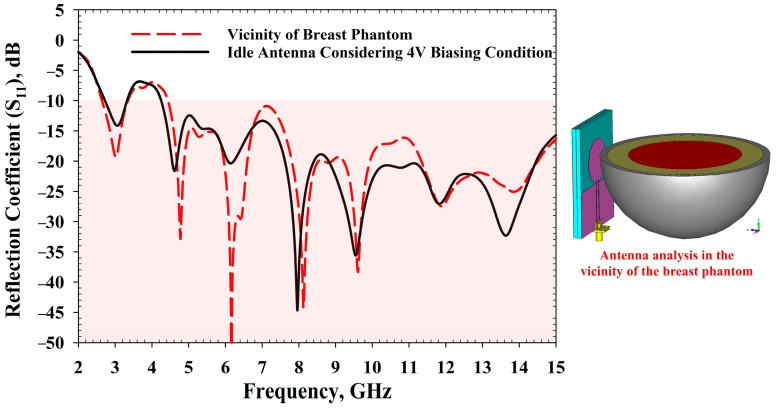
Effect of human tissue over antenna performance.

One of the significant drawbacks of the wearable antenna is the mutual coupling between the high-permittivity human body and the antenna [45,46]. Therefore, during the antenna design process, this factor was taken into account, as the antenna is intended to be placed very near to the human body. Figure 14 demonstrates how the *S*_11_ results were affected when the antenna was placed near the breast phantom. Nonetheless, the tunability feature of the antenna allows it to be retuned to the desired frequency region, even though results could be shifted to higher or lower frequencies.

## 4. Heterogeneous Breast Model Design

Prior to clinical trials, the utility of MWI breast cancer detection systems can be investigated using tissue mimicking materials, with several approaches used to build a realistic breast phantom [26]. As shown in Table 1, previous work in this field have used several approaches to fabricate a realistic breast phantom using tissue-mimicking materials. Nonetheless, there is still no consensus or standardisation in the field regarding the best version of lab-made breast phantom for studies on MWI systems. Therefore, one of the objectives of this study was to fabricate and experimentally validate the performance of the proposed MTM-based reconfigurable textile antenna with a realistic heterogeneous lab-made breast phantom.

This section focuses on the development of a breast phantom, involving simulation and fabrication procedures, using materials to mimic the heterogeneous breast tissue composition in vivo. In this study, the simulated and fabricated breast phantom was developed based on the dielectric properties listed in the Table 4.

**Figure 15:**
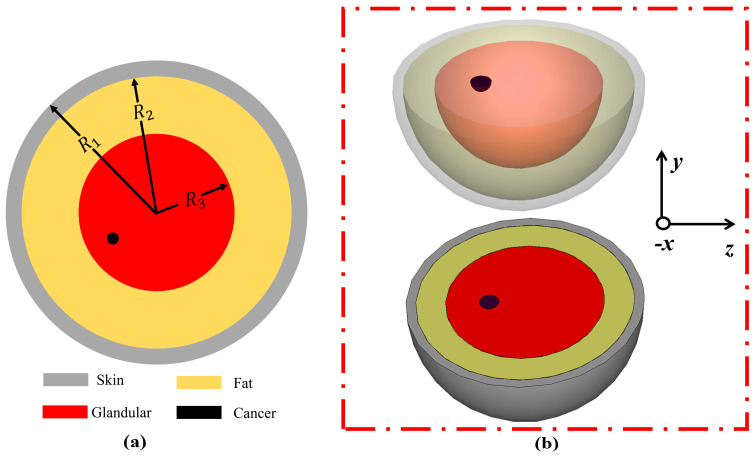
(**a**) Hemispherical breast model. (**b**) Simulated 3D breast phantom model in CST Studio Suite simulator.

Figure 15 exhibits the design of the heterogenous breast phantom model, which consists of three layers. Figure 15a shows a hemispherical breast model with a height of 60 m and *R_1_* = 60 mm, *R_2_* = 58 mm, and *R_3_* = 40 mm. Figure 15b presents the simulated breast phantom model in the CST MWS simulator for analysis. Furthermore, a cancer 10 mm in diameter was considered in both simulation and fabrication.

### Heterogenous Breast Phantom Fabrication

**Figure 16:**
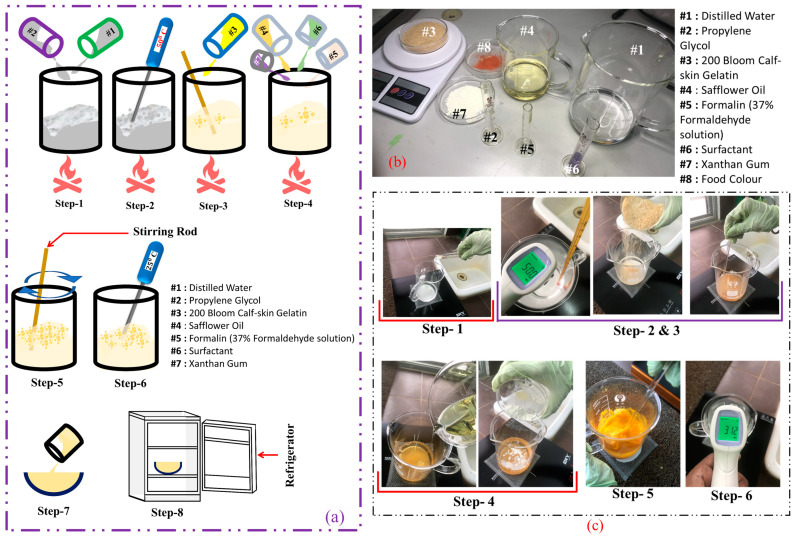
Heterogeneous breast phantom fabrication procedure: (**a**) schematic diagram of fabrication procedure; (**b**) photographic of different materials were used in fabrication processes; (**c**) all the steps were involved to carry out fabrication in laboratory environment.

Fabrication of each tissue type in the breast phantom has a common preparation phase with different material concentrations, as set out in Table 5. Figure 16 depicts the steps that involved fabrication of the breast phantom.

Fabrication of the breast phantom was performed using the following steps and strategies (Figure 16a).

**Step 1:** The measured amount of distilled water was mixed with propylene glycol in a beaker, placed over a heater with a wire gauze and ceramic centre.

**Steps 2 and 3:** Temperature of the mixture was monitored. At 50 °C, the 200 Bloom calf-skin gelatine was added and mixed properly until it was completely dissolved, resulting in a yellow mixture.

**Step 4:** As the gelatine was dissolving, the rest of the materials were added to the heated solution.

**Step 5:** The solution was removed from the heater and continuously stirred until it became cool. Stirring was carried out vigorously, taking care to avoid air bubbles in the mixture which may affect its dielectric properties.

**Steps 6, 7, and 8:** Once the mixture reached 25 °C, it was poured into a 3D-printed breast mould and then refrigerated overnight.

**Figure 17:**
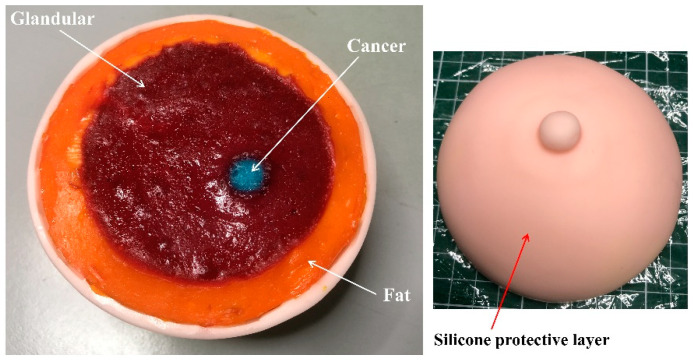
Heterogenous breast phantom prototype.

Figure 16b depicts the different materials used in fabrication processes, while Figure 16a illustrates the steps involved in fabrication of the breast phantom. To visually delineate the different tissue layers of the breast phantom and cancer, food colourings were used. Egg yellow was used for the fat layer, carmoisine for the glandular and skin layer, and true blue for the cancer. Then, 3D-printed moulds were used to shape the fabricated breast phantom. Figure 17 shows a heterogeneous breast phantom with a hemispherical shape of 60 mm in radius for this study. Furthermore, commercially available silicone breast pads with a thickness of 3 mm were utilised as a protective layer and to maintain the shape during further analysis. The purpose of adopting silicon breast pads was to analyse the microwave breast imaging in an optimal environment. Since silicone is a widely used material for clothes, it can help with integration of sensors/antennas for MWI.

**Figure 18:**
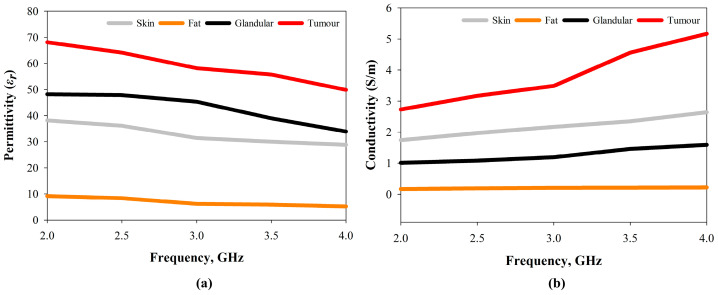
Dielectric properties of the fabricated heterogeneous breast phantom: (**a**) permittivity and (**b**) conductivity.

The measurements of dielectric constant were carried out within 2–4 GHz. A Keysight 85070E dielectric probe pack (Agilent Technologies, Bayan Lepas, Penang, Malaysia) equipped with an E5071C network analyser (Agilent Technologies, Bayan Lepas, Penang, Malaysia) was used. The probe test is one of the most common and simple techniques for determining dielectric properties [50]. First, three standards, namely open, short, and water, were used to calibrate the probe. Thereafter, the breast phantom’s layers were measured in the mentioned frequency range. To ensure that there was no distance between the probe and the sample, the exterior surface was sanded down. The measured dielectric/relative permittivity and conductivity over the frequency are presented in Figure 18a,b, respectively. The breast phantom skin layer dielectric constant was between 28 and 38, and the conductivity was between 1.74 and 2.64. The dielectric constant of fatty tissue was between 5.19 and 9.17, and the conductivity was between 0.17 and 0.22. The glandular tissue had a dielectric of 33–48 and a conductivity of 1.01–1.59, whereas the cancer tissue had the highest dielectric and conductivity at 49–68 and 2.73–5.17, respectively, since it contained more water. These results closely approximate previous measurements performed in vivo on real human breasts [50], suggesting that the fabricated breast phantom is well suited for studies using the MWI system.

## 5. Proposed MBRU Antenna-Based Microwave Imaging System

The DAS and DMAS microwave imaging algorithms, considered among the best imaging algorithms, were adopted for MWI [32]. This part of the study was carried out in three stages to detect the cancer in the breast phantom using backscattered signals. In stage 1, a microwave breast scan was performed involving collection of signals in the time and/or frequency domain. An Agilent E5071C Network Analyser (Agilent Technologies, Bayan Lepas, Penang, Malaysia) was used in a frequency-domain system to carry out a frequency scan within a defined frequency range. Data attained from stage 1 were further processed under stage 2 to create the dataset for further analysis. In stage 3, the microwave-based image was visualised with the help of the Microwave Radar Based Imaging Toolbox (MERIT) [51], using the DAS and DMAS imaging algorithms.

### 5.1. Simulation-Based Setup

**Figure 19:**
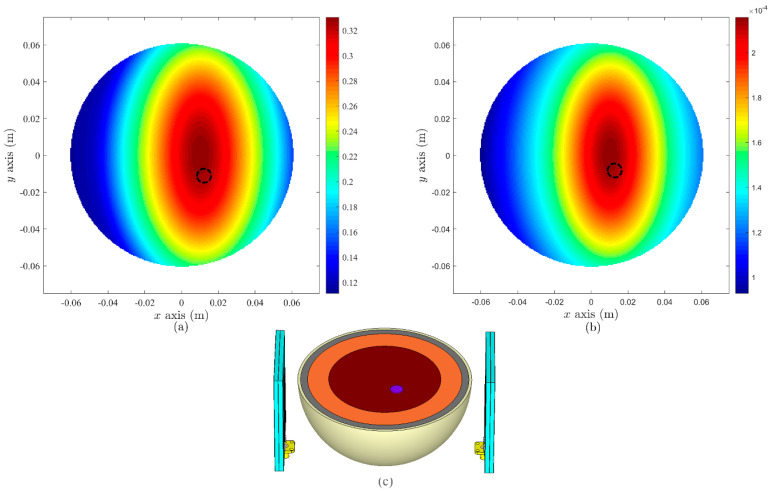
MWI results based on two antennas in simulation: (**a**) DAS, (**b**) DMAS algorithms, and (**c**) depiction of simulated setup.

**Figure 20:**
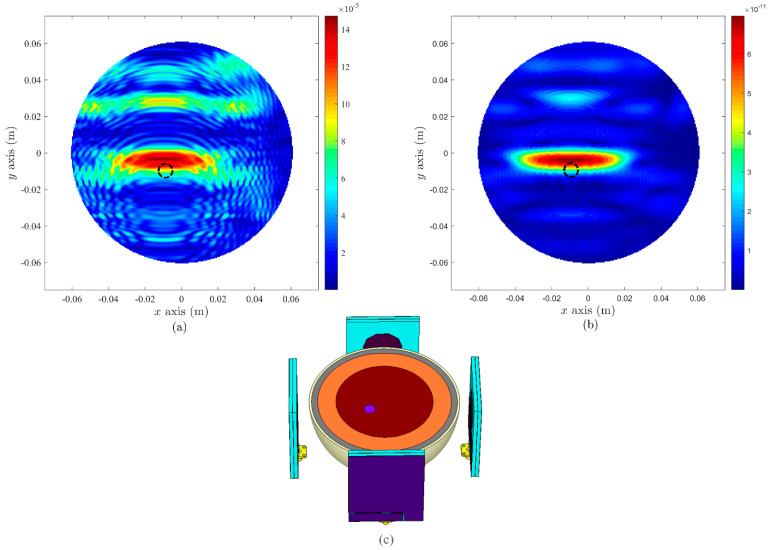
MWI results based on four antennas in simulation: (**a**) DAS, (**b**) DMAS algorithms, and (**c**) depiction of simulated setup.

As a part of WP4 (Figure 1), MERIT-based microwave imaging algorithms (using DAS and DMAS algorithms) were adopted in this study based on extracted data samples from simulation-based setup. The simulated breast phantom was irradiated with radio-frequency pulses from antennas placed at various positions around the breast phantom in a standard microwave breast imaging configuration, where each antenna functioned both as a transmitter (Tx) and as a receiver (Rx) in both simulation and experimental setup. The simulated breast phantom also included a 2 mm thick silicone layer, corresponding with the silicone protective layer of the fabricated breast phantom. Figure 19 depicts two antennas-based MWI simulated setup, while Figure 20 shows the simulated setup for four antennas. In both figures, a black dotted circle indicates the actual cancer location, where images were produced from the simulated breast phantoms that included a cancer. When number of antennas were increased from two to four, the quality of data and image were enhanced, suggesting that the signals generated from the antennas have sufficient depth of tissue penetration for cancer detection.

### 5.2. Experimental Setup

**Figure 21:**
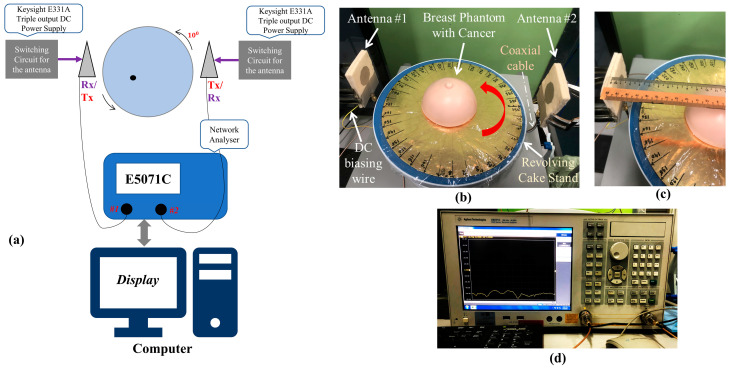
(**a**) Depiction of the data acquisition setup in this study, where the antennas are represented by the triangle shape surrounding the breast. Microwave imaging experimental setup: (**b**) photograph of MBRU-based setup; (**c**) maintaining the distance from antennas to breast phantom; (**d**) sample of backscattering data measurements.

As a part of WPs in WP6, the experimental MWI using fabricated breast phantom and the proposed antennas was performed, and data were collected using this setup, as depicted in Figure 21. The interference between the microwave signals from the antennas and the tissues of the breast results in the scattering signals which can be affected by the speed of path propagation, phase, polarisation, and strength of the surface [52]. In a multistatic setup, one antenna transmits, and several antennas receive for any given measurement, and all backscattered and transmitted signals are received in this sort of configuration. However, to attain optimum results using this multistatic antenna setup, the phantom was rotated at 10° in this study, as depicted in Figure 21a. To improve the quality of imaging, the number of antennas was increased, as was previously identified from the simulation studies. A ten-inch gold colour cake board was marked properly between 0 to 360 degrees, with steps of 10 degrees (Figure 21b), and then set on a cake rotating table. An eight (8) centimetre distance was maintained between each antenna and the breast phantom (Figure 21c). Then, the backscattering data were collected for further analysis.

**Figure 22:**
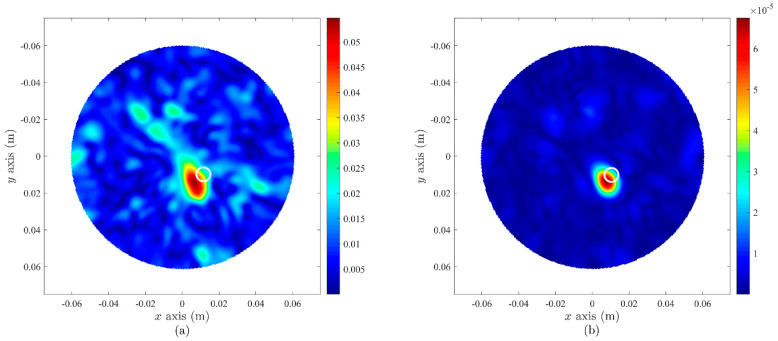
Microwave imaging results based on experimental setup using the proposed antenna and fabricated breast phantom considering a cancer inside. Result based on (**a**) DAS and (**b**) DMAS imaging algorithms.

The reconstructed image based on the experimental setup is presented in Figure 22, with the area of high contrast representing the centre of the cancer. However, the location of the cancer on the image was slightly off-centre from its actual location (represented by the white circle in each image). The image based on the DMAS algorithm gave a closer approximation to the actual cancer location compared to that using the DAS algorithm. Although the location of the cancer on the image was not precise, this issue can be resolved by increasing the sampling of backscattered data, since the image quality depends not only on quality of signal generated from the antennas but also the maximum number of data sampled.

## 6. Conclusions

We have described, for the first time, the design and development of an MTM-based directional CPW-fed frequency-reconfigurable textile antenna incorporated with RF varactor diodes for microwave imaging of breast cancer and validated this imaging system in a realistic breast phantom. Bandwidth improvement was achieved with the integration of the MTM into the antenna design and incorporation of an L-slot on its ground plane to facilitate the use of RF varactors to achieve frequency tunability. Both simulation and measurement proved that this antenna is capable of continuous frequency reconfiguration to distinct frequency bands between 2.42 GHz and 3.3 GHz with a frequency ratio of 2.33:1 and a static frequency band from 4 to 15 GHz. Frequency reconfigurability was performed using a simple biasing network consisting of four parallel RF varactor diodes located on the slotted ground plane. Besides the simplicity of fabrication, the antenna could be simply tuned by reverse bias voltage to its biasing circuit. Simulated and measured radiation patterns exhibited a good agreement with consistent directional patterns of radiation. Furthermore, SAR analysis in the vicinity of the breast phantom demonstrated that the antenna is compatible and safe for use on the human body, while the bending analysis showed minimal deformation effects. An MWI system using the MBRU antenna with DAS and DMAS algorithms was found to detect a cancer as small as 10 mm in a breast phantom, demonstrating the feasibility of imaging using the MBRU antenna. Although the results of the imaging experiments did not achieve a high accuracy of cancer localisation, increasing the sampling of data by increasing number of antennas may improve the accuracy. The MTM-based reconfigurable antenna can be efficiently tuned for MWI and potentially be used in a flexible/wearable format.

## Figures and Tables

**Table 1:** Comparison of MTM-based UWB antenna for breast cancer detection system.

Ref.	Antenna Type	Antenna Size (mm^2^)	Operating Frequency Range (GHz)	Tunability Feature	Frequency/Time Domain	Imaging Algorithms	Phantom and Cancer Object	Substrate Type
[26]	Index Near-Zero Metasurface Loaded antenna	77.72 × 60	2.7–8	No	Time and Frequency domain	Iterative Variant of Delay Multiply and Sum (IC-DMAS)	Lab-made realistic heterogeneous phantom with 1 and 2 cancer objects	Rigid
[27]	Slotted antipodal Vivaldi antenna	40 × 40	3.01–11	No	Time-domain	DMAS	Lab-made homogenous phantom with single cancer	Rigid
[28]	Antipodal Vivaldi Index	40 × 40	2.5–11	No	Frequency Domain	IC-CF-DAS	Lab-made homogenous phantom with multiple cancer	Rigid
[29]	Negative index (MTM loaded UWB antenna	27.5 × 19.4	2.97–15	No	Time and Frequency domain	DMAS	Lab-made realistic heterogeneous phantom with 2 cancer objects	Rigid
[30]	AMC based CPW-fed Microstrip antenna	76 × 78	3.1–7.6	No	–	–	Commercially available off self-breast phantom with single cancer	Rigid
[31]	Slotted patch	44 × 52.4	3.5–15	No	Frequency domain	Confocal Imaging	Simulated phantom	Rigid
This Work	SNG/NZRI metamaterial, L-shaped slot loaded with RF varactors	80 × 61	(2.42–3.3 GHz) ~reconfigurable with narrow band and (4–15 GHz) ~static bandwidth BW = 11.88	Yes	Time and Frequency domain	DAS and DMAS	Lab-made realistic heterogeneous phantom with 1 cancer objects	Flexible

**Table 2:** Parameters of the MTM unit cell.

Parameters	*a*	*b*	*c*	*d*	*e*	*f*	*g*_1_	*g*_2_	*g*_3_
Value (mm)	10	7.8	5.2	3.2	1.81	1.3	2.49	3	1.98

**Table 3:** Parameter dimensions of the MBRU antenna.

Para.	Value (mm)	Para.	Value (mm)	Para.	Value (mm)
*L*	80	*R*	33	*W*_1_	15.5
*W*	61	*G*_1_	0.5	*L*_1_	10.8
*W_f_*	3.85	*G*_2_	0.8	*W*_2_	28
*L*_1_	33.5	*h*	3	*L*_2_	8.31

**Table 4:** Dielectric properties of the breast tissues: fat, skin, glandular, and cancer at 3 GHz.

Reference	Fat		Skin		Glandular		Cancer	
	ε_r_	σ (S/m)	ε_r_	σ (S/m)	ε_r_	σ (S/m)	ε_r_	σ (S/m)
[47,48]	5	0.1	38	1.8	47	2.1	67	3.1

**Table 5:** Composition of different tissue layers in the breast phantom [49,50].

Materials	Quantity			
	Skin	Fat	Glandular	Cancer
Distilled water	80 mL	40 mL	80 mL	100 mL
Propylene glycol	7 mL	2 mL	7 mL	6.5 mL
200 Bloom calf-skin gelatine	5.88 g	7 g	5 g	9 g
Safflower Oil	14 mL	39 mL	21 mL	7 mL
Formalin (37% formaldehyde solution)	0.30 mL	0.30 mL	0.30 mL	0.30 mL
Surfactant	0.30 mL	0.30 mL	0.30 mL	0.30 mL
Xanthan gum	1.3 g	1.3 g	1.3 g	1.3 g

## Data Availability

The study did not report any data.
